# Retention in care for adolescents who were newly initiated on antiretroviral therapy in the Cape Metropole in South Africa

**DOI:** 10.4102/hivmed.v21i1.1077

**Published:** 2020-07-22

**Authors:** Brian van Wyk, Ebrahim Kriel, Ferdinand Mukumbang

**Affiliations:** 1 School of Public Health, Faculty of Community and Health Sciences, University of the Western Cape, Cape Town, South Africa

**Keywords:** HIV, AIDS, adolescents, youth, retention in care

## Abstract

**Background:**

Long-term retention of adolescents aged 10 -19 years on antiretroviral therapy (ART) is crucial to achieve viral load suppression. However, it is reported globally that adolescents have lower retention in care (RiC) on ART, compared with children and adults.

**Objectives:**

To determine the prevalence and predictors of RiC of adolescents over 2 years following initiation onto ART in public health facilities in the Metropole District Health Services of the Western Cape province in 2013.

**Methods:**

Data of 220 adolescent patients who were newly initiated on ART in 2013 were extracted from the provincial electronic database, and subjected to univariate and bivariate analyses using SPSS.

**Results:**

The rate of RiC post-initiation was low throughout the study period, that is, 68.6%, 50.5% and 36.4% at 4, 12 and 24 months, respectively. The corresponding post-initiation viral load suppression levels on ART of those remaining in care and who had viral loads monitored were 84.1%, 77.4% and 68.8% at 4, 12 and 24 months, respectively. Retention in care after initiation on ART was higher amongst younger adolescents (10-14 years), compared with older adolescents (15-19 years). Male adolescents were significantly more likely to be retained, compared with females. Pregnant adolescents were significantly less likely to be retained compared with those who were not pregnant.

**Conclusion:**

Key interventions are needed to motivate adolescents to remain in care, and to adhere to their treatment regimen to achieve the target of 90% viral load suppression, with specific emphasis on older and pregnant adolescents.

## Introduction

Globally, it has been estimated that 190 000 (59 000–380 000) adolescents, between the ages of 10 and 19 years, were newly infected with human immunodeficiency virus (HIV) in 2018, and the total number of adolescents living with HIV (ALWH) was 1.6 million (1.1–2.3 million), which accounts for 4% of all people living with HIV (PLWH).^1^ Sub-Saharan Africa has the highest number of HIV-infected adolescents with about 1.5 million of them. In South Africa (SA), it is estimated that 280 000 children aged between 0 and 14 years were living with HIV in 2017, and that just under 7 million persons aged ≥ 15 years were PLWH.^2^ The national HIV household survey estimated HIV prevalence in 0–14-year-olds to be 3.0% and 2.4% for females and males, respectively.^3^ In 15–19-year-olds, it was 5.8% in females and 4.7% in males. The number of adolescents aged 15–19 years receiving antiretroviral therapy (ART) in SA increased tenfold between 2005–2008 and 2013–2016.^4^ This increase is attributed to perinatally infected infants surviving into adolescence and to a rising incidence of HIV in behaviourally infected 15–19-year-olds.

Despite success in ART roll-out in most countries over the last decade, acquired immune deficiency syndrome (AIDS)-related deaths amongst adolescents have increased whilst declining in other age groups.^5^ To prevent AIDS-related deaths, the infected must be diagnosed, receive ART and remain in care to maintain viral load (VL) suppression. This would help achieve the 90-90-90 targets of the Joint United Nations Programme on HIV and AIDS (UNAIDS).^6^ Retention on ART is particularly challenging for key populations, such as adolescents, amongst others, and has been noted as a global priority for action.^7,8^ Previous studies confirm that adherence, retention in care (RiC) and treatment outcomes for adolescents in southern Africa are worse, compared with adults.^9,10,11^

However, routine monitoring of HIV treatment programmes does not report RiC and treatment outcomes for adolescents (10–19 years); they report only for children (0–14 years) and adults (15 years and older).^11^ In this way, adolescent problems with RiC and treatment outcomes remain undetected in the monitoring of HIV programmes. It is argued that because of the general adherence problems faced by adolescents globally, specific analysis is needed to report outcomes for this age group at a health-systems level.^8^

## Objectives

This article reports on the RiC of ALWH aged 10–19 years who were newly initiated on ART in public health facilities in the Western Cape Metropole, SA, in 2013, and the risk factors associated with remaining in care at 4, 12 and 24 months post-initiation on ART.

## Methods

### Design

We conducted a retrospective cohort analysis of adolescents aged 10–19 years, who were initiated on ART in 2013 in the Western Cape Metropole.

### Study context

The Western Cape’s ART programme has been in existence for over 10 years. The 2013 version of the ART guidelines was updated in 2016, and had consolidated adult, adolescent, paediatric and prevention of mother-to-child transmission of HIV guidelines.^12^ In the province of the Western Cape, most adults and children access ART services at primary healthcare facilities, such as clinics, community day centres and community health centres. At the end of June 2017, this province had 237 285 patients on ART, of whom 229 171 were aged ≥ 15 years and 8114 were aged < 15 years. The Cape Town Metropole accounts for 74.3% (167 833) of the total number of ART patients in the Western Cape, that is 162 092 aged ≥ 15 years and 5741 aged < 15 years.^13^ Antiretroviral therapy services are rendered at the various Western Cape facilities, as outlined in Table 1.^13^

**TABLE 1:** Facilities in the Western Cape rendering antiretroviral therapy services (*N* = 256).^13^

Facility type	Rural	Metro	Total
Clinic (including satellite clinics)	138	26	164
Mobile clinics	8	0	8
Community day centre	17	29	46
Community health centre	0	9	9
District hospital	4	6	10
TB hospitals	4	2	6
Regional hospitals	0	4	4
Correctional centres	8	1	9
**Total**	**179**	**77**	**256**

TB, tuberculosis.

However, services rendered at these facilities vary. Some provide only adult ART services. Others offer both adult and paediatric support. Some facilities offer ART daily, whilst others provide ART only on certain days of the week. Human resource capacity also varies. Not all facilities have resident medical officers or clinical nurse practitioners. Hence, the need for outreach support arises. The capacity to care for adults in the Western Cape has improved with the implementation of the nurse-initiated management of antiretroviral treatment (NIMART) programme, whereby nurses are trained and receive structured mentorship and accreditation to initiate first-line ART. A challenge in the rural areas is the irregular access to competent and skilled clinicians to manage paediatric patients and complicated adult and adolescent patients. As previously stated, most ART services are designated as paediatric or adult. Adolescent-specific ART services are not yet part of standard care being offered at all ART facilities. Those that do are limited to services initiated and/or supported by tertiary hospitals or non-profit organisations.

### Data source

Two data sources were used for this analysis: the Three Interlinked Electronic Registers.Net (TIER.Net),^14^ an electronic ART database developed by the University of Cape Town’s Centre for Infectious Disease Epidemiology and Research, and patients’ folders accessed at the treating facilities. Tier.Net is used operationally in the public health facilities of SA to monitor baseline clinical care and client outcomes over time. It is also the platform on which HIV tests are electronically captured in the public sector.^15^

We first visited the Tier.net platform to obtain data on all those who met the inclusion criteria. With the use of our data-capturing form, we searched for the relevant information from the Tier.net platform. Where information was missing, we accessed the patient’s folder to confirm the availability of the required information.

### Study participants

Data were found for 332 ALWH newly registered on ART from 29 facilities across the Western Cape Metropole and extracted from the provincial Tier.net register. Only 220 participants were included in the final analysis. Of the 112 excluded, for 68, folders could not be found in spite of making numerous attempts to trace these documents at the various facilities. Furthermore, 28 patients were incorrectly captured as new patients when they had been transferred in from other facilities; 4 patients were not adolescents, and their birth dates had been incorrectly captured; and 12 patients had been incorrectly captured as having initiated ART in 2013.

### Main outcome measures and analysis

We extracted data on sociodemographic characteristics (age, sex, source of income, type of dwelling, disclosure to significant other and reported alcohol or other drug use) and clinical characteristics (CD4 count, WHO stage, pregnancy and ART regimen).

Bivariate analysis was conducted to determine the significance and strength of association between RiC at 4, 12 and 24 months and various sociodemographic and clinical characteristics. Statistical significance was tested by using the chi-square test, with significance set at *p* < 0.05, and where significant, the strength of association was calculated as risk ratios (RRs) with 95% confidence interval (CI), using SPSS v23. Our use of 4, 12 and 24 months rather than 6, 12 and 24 months is informed by the operational guidelines of the Western Cape’s ART programme, which requires the first VL test to be conducted at 4 months and the patient to return for results in month 5.^16^ Patients would receive 1 month’s medication if they have unsuppressed VL, and 2 months’ supply if their VL is suppressed. The reason for the 4-month RiC measurement is to identify how many ALWH return for their VL tests. The subsequent RiC behaviour of the patients is measured annually.

Survival analysis was assessed with lost to follow-up (LTFU) as the outcome of interest. We did a comparative survival analysis for the age and sex of the study participants. We reported the hazard ratios and *p*-values. An ALWH was considered LTFU if they had not made contact with a treating healthcare facility within 90 days since their last registered contact for HIV-related treatment and care. The LTFU date was determined from the day when the patient was last seen at the clinic where they were provided with their last medication. Therefore, by using the intention-to-treat population in this study, the RiC definition was the proportion of HIV-infected adolescents alive and on ART at months 4, 12 and 24 in the entire study sample.

### Ethical consideration

The protocol was approved by the University of the Western Cape Biomedical Research Ethics Committee (Reference number: BM/17/1/15) and the Government Health Impact Assessment (Reference number: WC_2017RP58_418) Committee.

## Results

Of the 220 adolescents who were newly initiated on ART in 2013, the majority were ‘older’ adolescents, 15–19 years (*n* = 179, 81.4%) and female (*n* = 182, 82.7%) (Table 2). Most were financially supported by their families and friends (*n* = 129, 58.6%) and lived in a formal house (*n* = 116, 52.7%). As per HIV clinical treatment guidelines, the overwhelming majority (*n* = 182, 87%) had disclosed their HIV status to a significant other.

**TABLE 2:** Sociodemographic and clinical characteristics of adolescents retained in antiretroviral therapy at Metropole District Health Service facilities in the Western Cape, 2013–2015 (*N* = 220).

Characteristics	Total		Month 4			Month 12			Month 24		
	*n*	%	*n*	%	*p*	*n*	%	*p*	*n*	%	*p*
**Age**											
10–14 years	41	18.6	36	87.8	0.003*	33	80.5	< 0.001*	28	68.3	< 0.001*
15–19 years	179	81.4	115	64.2	-	78	43.6	-	52	29.1	-
**Sex**											
Male	38	17.3	32	84.2	0.023*	25	65.8	0.038*	20	52.6	0.022*
Female	182	82.7	119	65.4	-	86	47.3	-	60	33.0	-
**Source of income**											
Employed	15	9.4	12	80.0	0.063	6	40.0	0.077	5	33.3	0.052
Family and friends	129	80.6	80	62.0	-	60	46.5	-	41	31.8	-
Grant	16	10.0	14	87.5	-	12	75.0	-	10	62.5	-
**Type of dwelling**											
Informal dwelling	69	36.1	47	68.1	0.243	34	49.3	0.817	23	33.3	0.395
Formal house	116	60.7	76	65.5	-	54	46.6	-	39	33.6	-
Hostel	4	2.1	1	25.0	-	1	25.0	-	0	-	-
Other	2	1.1	2	100	-	1	50.0	-	0	-	-
**Disclosure†**											
Yes	182	87.1	127	69.8	0.272	97	53.3	0.008*	70	38.5	0.044*
No	27	12.9	16	59.3	-	7	25.9	-	5	18.5	-
**Alcohol/drug use**											
Yes	27	15	22	81.5	0.059	10	37.0	0.401	6	22.2	0.279
No	153	85	96	62.7	-	70	45.8	-	50	32.7	-
**CD4 count**											
< 200 cells/mm^3^	42	19.3	30	71.4	0.742	23	54.8	0.634	18	42.9	0.218
200–349 cells/mm^3^	109	50.0	77	70.6	-	57	52.3	-	43	39.4	-
≥ 350 cells/mm^3^	67	30.7	44	65.7	-	31	46.3	-	19	28.4	-
**WHO stage**											
I	99	46.5	59	59.6	0.021*	39	39.4	0.024*	24	24.2	0.008*
II	48	22.5	34	70.8	-	29	60.4	-	24	50.0	-
III	51	23.9	43	84.3	-	31	60.8	-	23	45.1	-
IV	15	7.0	10	66.7	-	9	60.0	-	6	40.0	-
**Pregnant**											
Yes	84	38.2	47	56.0	0.001*	30	35.7	0.001*	18	21.4	< 0.001*
No	136	61.8	104	76.5	-	81	59.6	-	62	45.6	-
**On IPT**											
Yes	15	6.8	9	60.0	0.346	7	46.7	0.407	4	26.7	0.658
No	165	73.2	111	67.3	-	80	48.5	-	60	36.4	-
**ART regimen**											
TFE	157	71.4	100	63.7	0.010*	65	41.4	< 0.001*	44	28.0	< 0.001*
T3E	27	12.3	17	63.0	-	15	55.6	-	10	37.0	-
A3E	34	15.5	32	94.1	-	29	85.3	-	25	73.5	-
Z3E	1	0.4	1	100	-	1	100	-	0	-	-
T3L/rit	1	0.4	1	100	-	1	100	-	1	100	-

ART, antiretroviral therapy; WHO, World Health Organization; IPT, isoniazid preventive therapy.

* , Statistical significance at *p* < 0.05.

† , Disclosure entailed adolescents disclosed to and those who disclosed.

TFE (Tenofovir + Emtricitabine + Efavirenz); T3E (Tenofovir + Lamivudine + Efavirenz); A3E (Abacavir + Lamivudine + Efavirenz); Z3E (Zidovudine + Lamivudine + Efavirenz); T3L/rit (Tenofovir + Lamivudine + Lopinovir/ritonavir).

The median CD4 count at ART initiation was 292.5 cells/mm^3^ (interquartile range [IQR]: 228.8–391.3). Only two participants had no baseline CD4 count recorded. Half of the participants (*n* = 109) were initiated with a CD4 count between 200 cells/mm^3^ and 349 cells/mm^3^, and 19% (*n* = 42) had a baseline CD4 count of < 200 cells/mm^3^ as per the HIV treatment guidelines of 2013.

Of the 213 participants who had WHO staging done at ART initiation, 46.5% and 22.5% were WHO stages I and II, respectively, and 23.2% and 6.8% were WHO stages III and IV, respectively. As with the CD4 counts, clinical staging is taken into consideration in the universal test and treat era.

**FIGURE 1:**
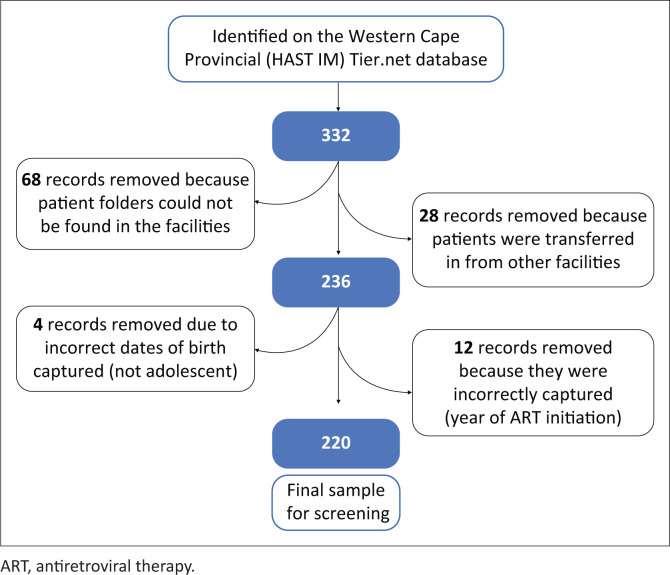
A consort diagram illustrating the sampling process.

Observed RiC was low throughout the study period with 68.6%, 50.5% and 36.4% adolescents being retained in care at 4, 12 and 24 months post-initiation on ART, respectively. Figure 1 illustrates the comparison of RiC at 4, 12 and 24 months between younger (10–14 years of age) and older (15–19 years of age) adolescents (90.2% vs. 63.7%, 82.9% vs. 43.0% and 68.3% vs. 29.1%, respectively). However, RiC of the younger adolescents at month 4 was just over 90%, but the younger adolescents at months 12 and 24 fell short of 90%. The older adolescents showed poorer rates of RiC at months 4, 12 and 24, compared with the younger adolescents at the same time periods.

Table 3 shows that a significantly higher number of younger adolescents (10–14 years) were retained in care at 4, 12 and 24 months post-initiation on ART, compared with older adolescents (15–19 years). At 4 months post-initiation on ART, younger adolescents had 37% higher risk (likelihood) of RiC (RR = 1.37, 95% CI: 1.17–1.60) compared with older adolescents. At 12 months post-initiation on ART, younger adolescents had 85% higher risk of RiC (RR = 1.85, 95% CI: 1.48–2.31), compared with older adolescents, and more than two times higher risk (RR = 2.35, 95% CI: 1.73–3.20) at 24 months.

**TABLE 3:** Rate of retention in care amongst adolescents initiated on antiretroviral therapy in 2013 in Metropole District Health Service facilities in the Western Cape (*N* = 220).

Characteristics	Month 4		Month 12		Month 24	
	RR	95% CI	RR	95% CI	RR	95% CI
**Age**						
10–14 years	1.37	1.17–1.60	1.85	1.48–2.31	2.35	1.73–3.20
15–19 years	1.00	-	1.00	-	1.00	-
**Sex**						
Male	1.29	1.08–1.53	1.39	1.06–1.83	1.60	1.11–2.30
Female	1.00	-	1.00	-	1.00	-
**Disclosure to significant other**						
Yes	1.18	0.85–1.63	2.06	1.07–3.95	2.08	0.92–4.68
No	1.00	-	1.00	-	1.00	-
**WHO stage**						
I	1.00	-	1.00	-	1.00	-
II	1.16	0.93–1.34	1.35	1.09–1.53	1.52	1.24–1.69
III	1.29	1.14–1.42	1.35	1.10–1.53	1.46	1.15–1.66
IV	1.11	0.68–1.40	1.34	0.94–1.60	1.39	0.77–1.70
**Pregnant**						
Yes	0.73	0.59–0.90	0.60	0.44–0.83	0.47	0.30–0.74
No	1.00	-	1.00	-	1.00	-

RR, risk ratio; CI, confidence interval; WHO, World Health Organization.

Male adolescents had higher rates of RiC post-initiation of ART at 4 months (RR = 1.29, 95% CI: 1.08–1.53), 12 months (RR = 1.39, 95% CI: 1.06–1.83) and 24 months (RR = 1.60, 95% CI: 1.11–2.30), compared with female adolescents.

Adolescents who were pregnant had significantly lower rates of RiC post-initiation of ART, compared with all other adolescents at 4 months (RR = 0.73, 95% CI: 0.59–0.90), 12 months (RR = 0.60, 95% CI: 0.44–0.83) and 24 months (RR = 0.47, 95% CI: 0.30–0.74).

Adolescents who disclosed their HIV status to a significant other were two times more likely to be retained in care at month 12 (RR = 2.06, 95% CI: 1.07–3.95) than adolescents who did not disclose to a significant other.

Adolescents classified as WHO stage I at ART initiation had significantly lower rates of RiC at 4 months post-initiation, compared with adolescents who were classified as WHO stage III (RR = 1.29, 95% CI: 1.14–1.42). Those classified as WHO stages II and IV also had better rates of RiC at month 4, compared with adolescents classified as WHO stage I at baseline, but these did not reach statistical significance. At 12 months post-initiation of ART, those who were at WHO stage II (RR = 1.35, 95% CI: 1.09–1.53) as well as WHO stage III (RR = 1.35, 95% CI: 1.10–1.53) at baseline had a 35% greater risk (likelihood) of RiC, compared with those who were WHO stage I at ART initiation. Adolescents who were at WHO stage I at baseline also showed significantly lower RiC rates at 24 months post-initiation of ART, compared with those who were at WHO stage II (RR = 1.52, 95% CI: 1.24–1.69) and WHO stage III (RR = 1.46, 95% CI: 1.15–1.66) at baseline. At 24 months post-initiation of ART, adolescents who were classified as WHO stage II (RR = 1.35, 95% CI: 1.09–1.53) and those who were WHO stage III (RR = 1.35, 95% CI: 1.10–1.53) at ART initiation had a 35% greater risk of RiC, compared with those who were WHO stage I at ART initiation. Those adolescents who were classified as WHO stage IV at ART initiation showed better RiC rates at months 4, 12 and 24 post-initiation, but none of these reached statistical significance.

**FIGURE 2:**
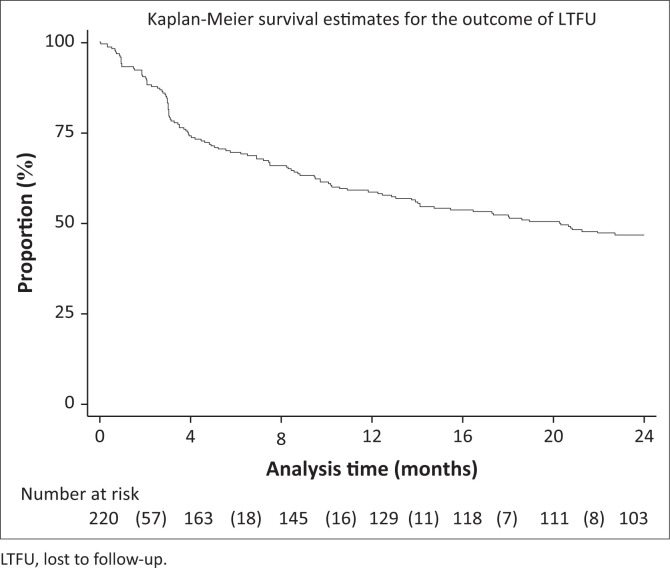
Overall rate of adolescents being lost to follow-up post-initiation of antiretroviral therapy.

**FIGURE 3:**
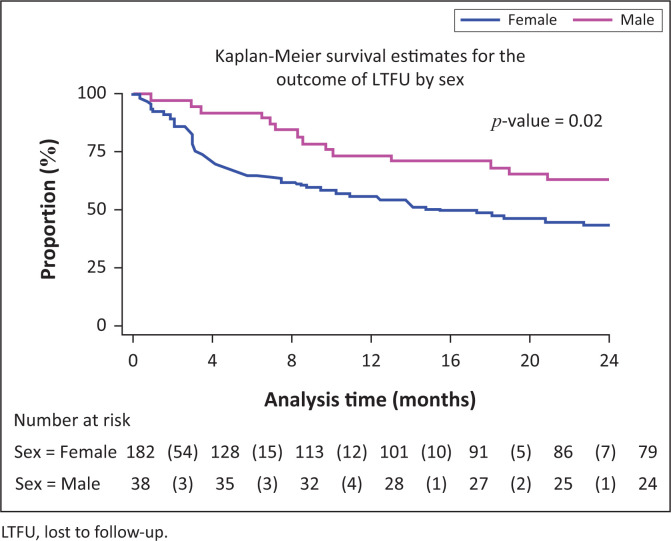
Rate of being lost to follow-up of adolescents post-initiation of antiretroviral therapy by sex.

**FIGURE 4:**
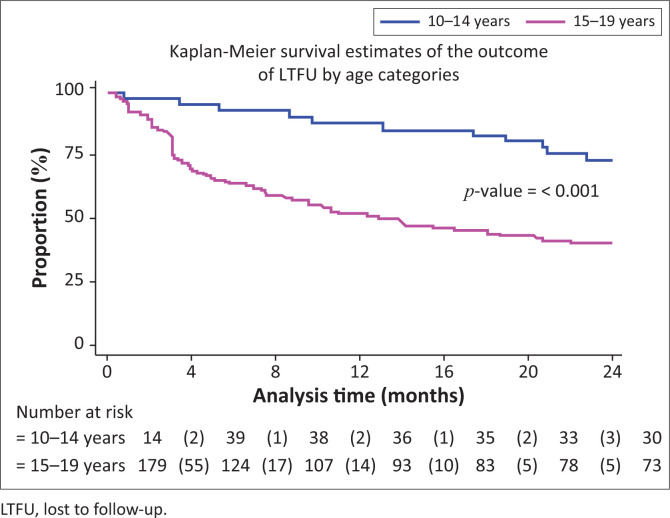
Rate of being lost to follow-up of adolescents’ post-initiation of antiretroviral therapy by age.

Figures 2, 3 and 4 show the survival curves of the study participants. The overall person-time at risk of being LTFU was 3303 months, with an incidence of 119/3807 (4/100) person-months. The hazard ratio for males compared with females was 0.71 (95% CI: 0.41–1.26). The hazard ratio for those aged 15–19 years compared with those aged 10–14 years was 2.53 (95% CI: 1.30–4.91).

## Discussion

In 2014, the UNAIDS set a global ART target of 90-90-90. This includes the goal that 90% of persons who test HIV-positive should be initiated on ART.^6^ Having successfully tested and initiated ALWH onto ART, their RiC and the maintenance of VL suppression of at least 90% are the ongoing challenges. Our study reports that the overall RiC of ALWH was low throughout the 24-month observation period. Contrary to our findings, Nabukeera-Barungi et al. found that 90.4% of Ugandan adolescents demonstrated good RiC with an LTFU of only 5%.^17^ A meta-analysis of six South African studies also reported a relatively high ALWH retention rate of 83% (95% CI: 68% – 94%) in the first 2 years on ART.^18^ The results of our study are reported with the intention-to-treat population as the denominator at every time point, that is, months 4, 12 and 24, and without the exclusion of transfer-outs, LTFU patients and the numerator being those alive and on ART. The above-mentioned studies did not measure RiC in the same manner. Nabukeera-Barungi et al.^17^ determined RiC by dividing those still active in care by the total number that started after subtracting those who died and transferred out from both the numerator and denominator.

Younger adolescents (10–14 years) demonstrated better RiC rates, compared with the older group. This observation could be attributed to the disproportionate attention offered to younger adolescents. In spite of the unique challenges posed by adolescents and ART, there is a dearth of comprehensive health services for adolescents, including interventions to improve RiC in sub-Saharan Africa.^19^ Nevertheless, younger adolescents show better RiC rates because they depend, to a greater extent, on their caregivers to handle their treatment journey. In this way, the RiC of the young adolescent is an extension of the dedication and understanding of the caregiver. Another study designed to investigate the RiC rates between younger and older adolescents in Zimbabwe demonstrated no differences in attrition amongst younger versus older adolescents.^20^

We found that older adolescents (15–19 years) were significantly less likely to be retained in care over the first 24 months, compared with younger adolescents. This finding is congruent with the trends reported in other studies^21,22^ and corresponds to the transition of adolescents from paediatric to adult HIV programmes – a known high-risk period for disengagement with care.^23,24,25^ Several authors have argued that patient-level challenges, such as developmental delays, mental health issues, stigma and social support at home and school, must be adequately addressed for a successful transition to take place.^26^ A supported transition requires a skilful adult treatment team and the provision of facilitated care aimed at overcoming the disruptions of the patient–paediatric provider relationship. The loss of ancillary support is required to foster independence, the exercise of autonomy and the growth of personal responsibility.^27,28^

Although male adolescents constituted a smaller proportion of the study sample, on average, they had greater RiC throughout the observation period, compared with females. Just under half of the female adolescents (*n* = 84/182, 46%) were initiated on ART whilst pregnant. They exited care at an alarming rate, that is, 44%, 64% and 79% at 4, 12 and 24 months, respectively. These findings correspond to those of Nuwagaba-Biribonwoha et al. who found a greater rate of LTFU amongst pregnant and non-pregnant female adolescents, compared with male adolescents.^29^ The current study reports lower RiC rates, compared with the 76.4% RiC at 12 months noted in a recent systematic review of pregnant and post-partum women in Africa.^30^ This report found younger age and same-day ART initiation to be risk factors for poor retention, as was initiating during pregnancy, particularly late pregnancy.

Our findings indicate that adolescents who were classified as WHO stage IV at ART initiation showed better RiC rates at months 4, 12 and 24 post-initiation, although no statistical significance was achieved. Individuals at WHO stages III and IV are likely to remain in care because they are motivated by their health status and by the association between treatment and health outcomes. Clinicians tend to monitor individuals who are at WHO stages III and IV more closely because of other comorbidities such as tuberculosis and other opportunistic infections requiring clinical assessments. However, Matyanga et al. found that a low CD4 count and advanced HIV infection at initiation were associated with LTFU.^20^ We also found that adolescents classified as WHO stage I at ART initiation had significantly lower rates of RiC at 4 months post-initiation versus those with a WHO stage III. Contrary to the results in our study, another Ugandan study found that the risk of LTFU of adolescents at 12 months was significantly greater amongst those on WHO clinical stages III and IV, compared with those on WHO stages I and II.^31^ People living with HIV at WHO stage I hardly display signs and symptoms associated with AIDS. The literature has attributed this low RiC behaviour amongst adolescents at stage I to not feeling ‘sick’ or feeling ‘well’ as a proxy of nothing being wrong.

Although the primary focus of our study was not on pregnant, HIV-infected adolescents, many in this sub-group were captured in our sample. This could be explained by the fact that pregnant, HIV-infected adolescents are often horizontally infected and receive their positive HIV test result for the first time when booking for antenatal care. Although vertical transmission of HIV is common amongst younger ALWH, horizontal transmission is a frequent mode of transmission in older adolescents. Adolescent boys tend to not access HIV treatment because they mostly remain asymptomatic at this stage.

Interventions such as task shifting, community-based adherence support, mHealth platforms and group adherence counselling emerged as strategies in adult populations that could be adapted for adolescents.^32,33^ These interventions may benefit older adolescents, especially those transitioning to adult programmes that utilise them. However, the effectiveness of, for example, ‘teen clubs’, has had mixed results. MacKenzie et al.^34^ reported that Malawian ALWH who were not in a teen club were less likely to be retained than those in teen clubs. On the other hand, Munyayi and van Wyk^35^ found that group-based adherence interventions such as teen clubs did not improve retention rates for younger adolescents in specialised paediatric ART clinics in Namibia but did hold potential for improving rates in older adolescents. Adolescent-only clinics and monthly meetings have been shown to improve the RiC of adolescents.^36^ To this end, we support the calls of other authors for interventions, especially targeting older adolescents whose needs are increased during the transition period.^23^

## Conclusion

Our study highlights low RiC for adolescents over the first 2 years after initiation on ART. Critical intervention is needed to motivate adolescents to remain in care, adhere to treatment and ultimately to achieve and maintain VL suppression (even when they are not feeling sick). Targeted interventions to address transition coordination – pre- and post-transition from paediatric to adult HIV programmes – are needed to counter older adolescents dropping out of care. Female adolescents who initiate ART whilst pregnant should receive special attention. This aligns with the increased need to provide and integrate appropriate sexual reproductive health services to ALWH. Behavioural interventions to improve adherence and RiC should ideally be embedded in community health services so that this forms part of an ‘extended’ HIV treatment package.
